# Influence of collection site on cerebrospinal fluid test results in horses with equine protozoal myeloencephalitis

**DOI:** 10.1093/jvimsj/aalag135

**Published:** 2026-07-08

**Authors:** Martin O Furr

**Affiliations:** College of Veterinary Medicine, Oklahoma State University, Stillwater OK 74078, United States

**Keywords:** antibody index, cerebrospinal fluid, equine, *Sarcocystis neurona*

## Abstract

**Background:**

Cerebrospinal fluid (CSF) analysis is a critical component in the diagnosis of equine protozoal myeloencephalitis (EPM). Test results obtained may differ based on different collection sites.

**Hypothesis/Objectives:**

Compare results of EPM-specific diagnostic tests obtained from different collections sites in EPM-affected and non-EPM affected horses.

**Animals:**

Twenty control and 7 EPM-affected horses.

**Methods:**

Prospective observational study. CSF was collected from the lumbosacral (LS) and cranial sites (atlantooccipital or C_1-2_) in 27 horses and EPM-specific diagnostic tests were performed. Data were summarized by collection site and disease status. Results were compared by Wilcoxon signed rank test in the non-EPM and EPM-affected cohorts.

**Results:**

The EPM-specific diagnostic test results (anti-*Sarcocystis neurona* CSF antibody titer, serum/CSF titer ratios, and *S. neurona* antibody index [AI]) in non-EPM affected horses did not differ based on collection site. In EPM-affected horses the median [interquartile range] CSF anti-*S. neurona* antibody titers were higher in samples from the LS site compared with fluid collected from the cranial sites (160 [1240] vs. 80 [600]; Wald test statistic, 21.0; *P* = .03) and the median serum/CSF titer ratio was also lower in fluid collected from the LS site (25 [46.8] vs 50 [93.7]; Wald test statistic, 0; *P* = .03). Median AI values did not differ based on CSF collection site.

**Conclusions and clinical importance:**

Results of EPM diagnostic tests on CSF may differ based on collection site and lead to misdiagnosis. The AI appears to be less affected by collection site than antibody titers or the serum/CSF titer ratio.

## Introduction

Collection of cerebrospinal fluid (CSF) for analysis is a foundational component of the diagnostic evaluation of horses with neurologic disease. Numerous studies have described the various techniques and collection sites that may be utilized, as well as the characteristics of the fluid in normal and diseased horses.^1–4^ Possible collection sites include the lumbosacral (LS), atlantooccipital (AO), and C_1-2_ site. Each site has advantages and disadvantages, and clinicians must consider these when selecting the collection procedure. A basic principle is to collect fluid from the location that is closest and caudal to the neuroanatomic location of the lesion (for unifocal disease),^5^ because of the bulk cranial-to-caudal flow of CSF.

Collection and analysis of CSF is a critical component in the diagnosis of equine protozoal myeloencephalitis (EPM), in which determination of the CSF anti-*Sarcocystis neurona* antibody titer is a key factor. The ratio of the serum reciprocal anti-*S. neurona* antibody titer to the corresponding CSF reciprocal anti-*S. neurona* antibody titer (hereafter referred to as titer ratio) is used as a proxy for the detection of intrathecal anti-*S. neurona* antibody production, for which a titer ratio <100 has been demonstrated to have high diagnostic value.^6^ A more precise estimation of intrathecal antibody production is the antibody index (AI), which is a calculated value derived from the serum and CSF reciprocal antibody titers, and the serum and CSF albumin concentrations.^7^ The known differences in CSF protein concentration based on collection site may lead to differences in EPM test results that might influence test interpretation. Although no difference has been reported in anti-*S. neurona* antibody titers or titer ratios when comparing LS to AO collection site in normal horses,^8^ this possibility has not been evaluated in horses with EPM.

The purpose of our study was to determine if diagnostic tests for EPM determined from CSF would differ based on the CSF collection site. The hypothesis tested was that the CSF reciprocal anti-*S. neurona* antibody titer, titer ratio and AI would not differ based on collection site in EPM-affected horses. The study protocol was approved by the Oklahoma State University Institutional Animal Care and Use Committee.

## Materials and methods

### Animals

The study consisted of 2 groups of horses: (1) horses with a clinical diagnosis of EPM (cases) or (2) non-EPM affected horses (controls). Horses used were obtained by donation after a public call for EPM-affected (or suspect) horses, as well as university-owned animals. Cases were defined as horses with neurologic abnormalities consistent with EPM, and that had a *S. neurona* titer ratio of <100 from the LS collection site. The titer ratio was used as the primary means of classifying cases and controls because it is a method commonly employed by clinicians in the field.

### Examination and sample collection

Routine physical and nervous system examinations were performed on all horses. Ataxia was scored using a modification of the Mayhew scoring system.^9^^,^^10^

After sample collection and analysis (described in Appendix 1) necropsy was performed by the pathology service of the Oklahoma Animal Disease Diagnostic Laboratory, limited to the central nervous system. Central nervous system tissue was harvested in a consistent manner from the cerebrum (bilateral), cerebellum, brainstem, and cervical, thoracic, and lumbar spinal cord segments as well as from any additional location based on the neurologic examination findings or abnormal regions identified by the pathologist at the time of tissue processing. One horse was examined and euthanized in Kentucky, and necropsy was performed at the University of Kentucky Veterinary Diagnostic Laboratory.

### Statistical analysis

Data were recorded in a spreadsheet and then transferred to a desktop statistical program (SPSS v27; IBM Corp, Armonk NY) for analysis. Normality of continuous data was evaluated by examination of the distribution, as well as the results of the Shapiro–Wilk test. CSF albumin and total protein concentrations and CSF cell counts were not normally distributed and were reported as median and interquartile range [IQR] as were reciprocal titers, titer ratios and *S. neurona* AI. Differences between cranial and LS results were determined using the Wilcoxon signed-sank test. An a priori *P*-value for significance of <.05 was determined and all analyses were 2-tailed. Proportions were compared using Fisher’s exact test. For purposes of statistical analysis and comparison, the initial diagnostic classification was based on LS test results.

## Results

Twenty-seven horses were utilized in the study, consisting of 20 control and 7 EPM-affected horses. Nineteen were geldings and 8 were mares, and multiple breeds were represented (1 Fox Trotter,1 Morgan, 1 Paso Fino, 4 Paint horses, 15 Quarter Horses, and 5 Thoroughbreds). The average age (±1 SD) of EPM horses was 16 ± 6.0 whereas the control horses averaged 17.2 ± 5.3 years.

Seven horses met the criteria for a diagnosis of EPM; necropsy examination did not indicate evidence of other disease and organisms were not detected on histopathologic examination in any horse. Two horses with a diagnosis of EPM were not euthanized but rather treated with a 1-month course of ponazuril (Marquis 15%; Boehringer Ingelheim, Duluth GA); both responded well, returned to normal, and remained normal for the ensuing 12 months after completion of treatment. Clinical abnormalities and pathologic findings in the EPM horses are presented in Table S1. No *S. neurona* organisms were observed in any horse.

Many different clinical abnormalities were detected in the control animals. Mild ataxia was recognized in 9 horses, historical seizures were reported in 2, and in another 5 horses musculoskeletal problems but no neurologic abnormalities were found; the remainder had no clinical abnormalities. Among control horses, pathologic examination identified severe cervical process joint arthritis in 2, non-specific axonal degeneration in 5, and areas of leptomeningeal mineralization in 2. Four control horses were considered to be clinically normal and a necropsy was not performed. Cerebrospinal fluid was collected routinely in all cases with the exception of one horse (Horse 5), in which only a small amount of fluid could be collected from the LS space, and total protein concentration and cell counts were not available for analysis for that horse.

### Cerebrospinal fluid analysis

Results of CSF total protein and albumin concentrations are summarized in Table 1. Individual differences for CSF total protein and albumin concentrations varied substantially by horse, with the differences between LS and cranial results indicating that the cranial concentrations were sometimes higher than the corresponding LS results. Among control horses, 10/20 LS CSF total protein concentrations were lower than the corresponding cranial result whereas the LS-cranial difference in CSF albumin concentration was positive in all cases. In EPM cases, only 1/6 horses had a lower LS total protein concentration than that of the cranial site, and the difference was always positive for CSF albumin.

**Table 1:** Summary of cerebrospinal fluid clinicopathologic values from 27 horses following collection from the cranial and the lumbosacral (LS) collection sites.

	**Control (*n* = 20)**			**EPM (*n* = 7)**		
	**Median (IQR)**		***P*** **Test statistic**	**Median (IQR)**		***P*** **Test** **statistic**
	**CRAN**	**LS**		**CRAN**	**LS**	
**CSF albumin** **(mg/dL)**	35.0 (23.5)	37.8 (17.9)	.33 71	44.5 (30.2)	54.8 (52.0)	.02 28
**CSF total protein** **(mg/dL)**	50.0 (38.0)	51.8 (19.0)	.81 91	46.0 (38.1)	72.1 (146.8)	.05 20
	**LS to CRAN difference**			**LS to CRAN difference**		
**CSF albumin**	−50.4 to +8.5			3.8 to 93.0		
**CSF total protein**	−33.0 to +12.5			−1.0 to 198.0		

Comparisons were tested using Wilcoxon signed rank test. Abbreviations: CRAN = atlantooccipital and C_1-2_ collection sites collectively; LS = lumbosacral.

Cell counts for samples collected from the cranial site for cases and controls were all within normal limits; 1 horse had blood contamination from the LS site. No significant differences were found between total nucleated cell count, red blood cell count or lymphocyte percentage between the cranial and LS collection sites for either control group horses or EPM cases. Results of cell counts for CSF are reported by individual in Table S2.

### Equine protozoal myeloencephalitis-specific tests

Group values for EPM-specific results are summarized in Table 2 and detailed by individual horse in Table S3**.** In control horses, no difference in results was found between the cranial and LS collection sites for the CSF anti-*S. neurona* titers, titer ratios, or *S. neurona* AI. In horses with a diagnosis of EPM, the LS CSF anti-*S. neurona* titer was significantly higher than from the cranial collection sites. The *S. neurona* titer ratio was lower in CSF collected from the LS site, and the *S. neurona* AI did not differ between the 2 collection sites. Serum anti-*S. neurona* titers did not differ between control and EPM-affected horses (1000 [IQR, 1500] vs. 4000 [IQR, 3000]; test statistic = 44.0; *P* = .16).

**Table 2:** Summary of EPM-specific diagnostic test results in 27 horses following paired lumbosacral and cranial site cerebrospinal fluid collection and assay.

	**Control (*n* = 20)**			**EPM cases (*n* = 7)**		
	**Median (IQ range)**		**Test statistic** ***P***	**Median (IQ range)**		**Test statistic** ***P***
**Serum titer**	1000 (1500)			4000 (3000)		44.0 .16
	**CRAN**	**LS**		**CRAN**	**LS**	
**CSF SAG2 4/3 titer**	5.0 (10.0)	5.0 (7.5)	10.5 .55	80 (600)	160 (1240)	21.0 .03
**SAG2 4/3 ratio**	200 (200)	200 (250)	5.0 .06	50 (93.7)	25 (46.8)	000 .03
**CSF antibody index**	0.55 (0.62)	0.54 (0.39)	62.5 .89	4.6 (14.1)	4.4 (32.0)	23.0 .13

Abbreviations: CRAN = cranial collection sites of C1-C2 and atlantooccipital; LS = lumbosacral collection site.

Individual comparisons in EPM-specific diagnostic tests are summarized in Table 3 and demonstrate the variability in titer results within individual animals. Among control horses, the results for CSF anti-*S. neurona* titer from cranial collection sites (3/20; 15%) were lower than results obtained from the corresponding LS space, whereas 4 of 20 (20%) were higher. In contrast in 6 of 7 (85%) EPM cases, the cranial anti-*S. neurona* titer was lower than the corresponding LS result. LS reciprocal CSF anti-*S. neurona* titers were twice those of the corresponding cranial sites in 4 horses, 4 times higher in 2, and the same in 1 horse.

**Table 3:** Summary of individual animal results comparing values obtained from cranial collection sites (atlantooccipital and C_1-2_ site) to the results from the lumbosacral (LS) space of the same horse.

	**Controls (*n* = 20)**				**EPM Cases (*n* = 7)**		
	**Lower**	**Equal**	**Higher**	***P***	**Lower**	**Equal**	**Higher**
**CSF titers cranial**	3 (15%)	13(65%)	4 (20%)	.005	6 (86%)	1 (14%)	0 (0%)
**Serum/CSF titer ratio cranial**	6 (30%)	13(65%)	1 (5%)	<.001	0 (0%)	1 (14%)	6 (86%)
**Antibody index cranial**	8 (40%)	5 (25%)	7(35%)	.269	5 (71%)	0 (0%)	2 (29%)

Data were compared using Fisher’s exact test. “Lower” indicates that the cranial result was less than the LS result from the same horse, “Higher” indicates the cranial result was larger than the corresponding LS result, and “Equal” indicates that the 2 results (cranial vs LS) were the same. Results indicate greater uniformity of results in non-EPM control horses.

Based upon inclusion criteria, 7 horses were classified as EPM cases. Using the same test performed on CSF from the cranial collection sites would have resulted in 5 of these 7 horses receiving a diagnosis of EPM and 2 horses an equivocal result of “100.” Of these 2 horses, the LS titer ratio was 25 and the LS site AI was 1.3 for 1 horse (Horse 4); the LS titer ratio and AI were 50 and 0.83, respectively, for the other (Horse 1). The AI from the cranial sites was negative (ie, < 1) for both horses (0.98 and 0.67, respectively). Among control horses, 3 had equivocal *S. neurona* titer ratios (ie, 100), and 2 of the 3 had the same result from the cranial collection sites, whereas the other had a value of 200 from the cranial sites. For these 3 control horses, the AI results were negative (ie, <1) for all 3 horses from both collection sites. For non-EPM affected horses using the titer ratio from either site would not have resulted in any misclassifications, but for EPM-affected horses using the titer ratio from the cranial site potentially could have misclassified 2 of 7 horses, depending upon the clinician’s interpretation of a test result of 100.

## Discussion

Our results confirm and extend previous findings regarding the differences between CSF clinical pathology variables of CSF collected from cranial sites (AO or C_1-2_) compared with fluid obtained from the LS site. Our study extends previous finding by demonstrating that CSF diagnostic results in EPM affected horses differ based on collection site.

Cerebrospinal fluid is continuously produced as an ultrafiltrate of plasma in the choroid plexus. Because of the fluid hydrostatic pressure differential, arterial pulsations and the influence of gravity, CSF flows cranial to caudal. Associated with this cranial to caudal flow along the neuraxis the total protein and albumin concentrations tend to increase.^11^ The physiological mechanism for the difference in CSF total protein concentrations along the neuraxis are explained by the decrease in CSF flow rate in caudal segments, with molecular flux as determined by Fick’s second law of diffusion.^12^^,^^13^ This flow rate also is influenced by the animal’s age, body weight, and presence of obesity, although these observations have not been specifically evaluated in horses.^14^^,^^15^ Another factor that may influence this finding is that the cranial to caudal flow is bulk, not laminar (ie, that it flows caudally unmixed). Flexion and extension movements of the spine and pulsations of intrathecal arteries lead to local currents and mixing of the CSF.^11^ In addition, horses spend a substantial amount of time with their heads down grazing, potentially influencing the gravitational influence on CSF flow and mixing. Although it has been suggested that these factors result in sufficient admixing of CSF that only minor differences are likely in samples collected from different sites,^16^ that conclusion was not unequivocally supported by our findings in horses with EPM using EPM-specific diagnostic tests. Although the *S. neurona* titer ratio does differ by collection site (cranial vs caudal), the AI index does not appear to be affected. Not enough horses with cerebral or brainstem lesions were available to comment on the supposition that a cranial lesion would result in a higher cranial CSF collection site *S. neurona* titer ratio than the LS site.

Previous studies have found that the total protein concentration is higher in CSF collected from the LS site compared with more cranial sites. One somewhat unexpected finding in our study was the frequency with which the total protein and albumin concentrations were higher in CSF collected from the cranial sites compared with the LS site. In a previous study, no group mean difference in total protein concentration was found between the 2 collection sites^2^ whereas another study reported that LS total protein concentration was slightly higher than the corresponding AO total protein concentration (52.1 mg/dL vs 49.0 mg/dL; *P* = .03).^8^ Other studies have reported variable results, sometime indicating higher LS total protein or albumin concentrations.^17^^,^^18^

Previous studies most often have been completed in clinically normal horses, and the influence of disease of the CNS may influence results. Observations about site-specific alterations in total protein and albumin concentrations raise questions regarding the interpretation of diagnostic assays that may be influenced by protein concentration, such as antibody titers or tests such as the AI that are calculated using CSF albumin concentrations.

 Reports of normal or non-EPM affected horses found that CSF anti-*S. neurona* antibody titers did not differ based on CSF collection site (AO vs LS).^8^ This finding in non-EPM affected horses was confirmed in our study, but in EPM-affected horses the LS anti-*S. neurona* SAG2,4/3 antibody titers were higher than the corresponding cranial site result. The median LS titer ratio however was not different than the cranial site titer ratio, and both met the established clinical diagnostic cutoff of <100.^6^ Based on our results, using the titer ratio alone in 2 of 7 cases might have led to a different diagnostic decision using the cranial rather than the LS site results. Both of these horses had a cranial collection site titer ratio of 100 which is an equivocal result that may be interpreted inconsistently in the field. Furthermore, it is possible that one or both of these horses may have been misclassified as EPM cases, without the gold standard of histologic confirmation of the organism in the central nervous system. A definitive conclusion remains unclear, but this situation highlights the challenges of EPM diagnosis and further demonstrates the role that CSF collection site may play. Ideally, a titer ratio result of 100 from either collection site should prompt further assessment using a more specific method (ie, AI).

The titer ratio is a proxy for a more precise assessment of intrathecal antibody production, in which a value greater than 1 indicates antigen-specific intrathecal antibody production.^7^^,^^19^ The AI is a calculated value comprised of the serum and CSF reciprocal antibody titers, and the serum and CSF albumin concentrations.^7^^,^^19^ Hence, alterations in CSF albumin concentration or CSF titers will have substantial impact on the final calculated value. As expected, the *S. neurona* AI in all non-EPM affected horses was below 1 (median 0.54 for LS and 0.55 for AO) and did not differ based on CSF collection site. Also, no difference in *S. neurona* AI was noted in EPM-affected horses based on collection site. In one EPM case (Horse 1; LS titer ratio, 50; AO ratio, 100) the LS *S. neurona* AI was 0.83 and the AO was 0.67, suggesting the LS titer ratio had resulted in a misclassification. A different clinical interpretation might have been made had the only test result been from a cranial sample, or if the AI results were prioritized in clinical decisions. Horse 4 also had atypical responses in that the LS AI was weakly positive (1.3) whereas the cranial AI was 0.98, which would be considered normal. The cranial titer ratio in Horse 4 was 100 whereas the LS result was strongly positive (25). Hence, it would be expected that the LS AI would be positive but perhaps would be expected to be larger than observed. The 2 major determinants of the AI are the CSF albumin concentration and the CSF reciprocal antibody titer. There was substantial blood contamination of the CSF from the LS collection in this horse (27 770 RBC/μL), which potentially influenced both of these results. Red cell counts up to 10 000 cells/μL have no effect on CSF antibody titers, and an effect on CSF antibody titer was only observed at values of 100 000 RBC/μL.^7^ In addition, RBC contamination greater than 10 000 cells/μL may increase CSF albumin concentration, which was observed in Horse 4. If other factors are unchanged increasing CSF albumin concentration will bias the AI calculation lower. In Horse 4 the LS CSF titer was 4× that of the AO CSF, which was also observed in another horse in the study that had no blood contamination; it does not therefore seem to be an outlier. Collectively, these observations suggest that blood contamination might have influenced the CSF results but likely minimally and it is not possible from the available data to conclusively determine the magnitude of the effect.

No difference was noted in cell counts between the LS and cranial collection sites in control horses, an expected finding that has been reported previously.^8^^,^^18^ Specific disease conditions likely affect this finding, because it has been reported in humans with leptomeningeal tumors^20^ (using paired samples) and in dogs with neoplastic or compressive diseases of the central nervous system^5^ (using unpaired samples) that cell counts differed based on collections site. In a study of dogs with corticosteroid-responsive meningitis, however, it was reported that total nucleated cell counts did not differ based upon collection site.^21^ Typically, EPM does not result in changes in the CSF white blood cell count,^22^ an observation that was also noted in the our study. Collectively, these observations suggest that the difference or lack of difference in total nucleated cell count based on collection site is influenced by disease pathophysiology. Case numbers in our study are too low to support any conclusion regarding EPM in horses however.

A limitation of this study is the small number of EPM-affected horses. Obtaining horses with confirmed EPM for donation proved challenging, and performing duplicate invasive procedures on client-owned clinical horses was not considered acceptable and hence not pursued. Although statistical significance was achieved even with the small number of EPM cases, study power was low and it is not possible to apply these results to the broader population of horses with EPM. Nevertheless, the recommendation to use a consistent collection site and pursue more specific testing (AI) in equivocal cases could be considered as best practice. An additional consideration is that the LS collection may have influenced the cranial collection sites results, because it was performed afterward, and this factor could have been addressed by randomizing the horses to collection order, which was not done because it would have required general anesthesia for the AO collection. Also, it would have been performed cranial to the LS site and potentially could have affected the LS CSF results. Furthermore, some horses would then have had to undergo anesthetic recovery only to be euthanized after the standing LS collection. The LS collection could have been performed during anesthesia, but in our experience the risk of blood contamination is higher with the horse in lateral recumbency. Given that the AO collection was performed within minutes of the LS procedure, in addition to the cranial to caudal flow of CSF, the potential for any effects on the AO CSF were considered unlikely, and hence randomization was not done.

Results of our study indicate that the site of CSF collection may influence the resulting *S. neurona* SAG2 4/3 titer ratios in horses with EPM, and that comparison of results obtained from cranial and LS collections sites are not valid. Equivocal results of the titer ratio should be further evaluated using the AI because it appears to be much more resistant to the effects of collection site.

## Supplementary Material

Supplementary_material_aalag135

## Off-label antimicrobial declaration

Authors declare no off-label use of antimicrobials.

## Institutional animal care and use committee or other approval declaration

Approved by Oklahoma State University Institutional Care and Use Committee (IACUC) Approval Number (ACUP)—IACUC-19-40.

## Appendix 1. Sample collection and processing

Whole blood was collected by jugular venipuncture into EDTA-coated vacutainer tubes (Vacutainer; Becton-Dickenson, Franklin Lakes, NJ) for determination of CBC at the clinical pathology laboratory of the Oklahoma State University Veterinary Teaching Hospital. Complete blood cell counts were performed using routine automated methods, and counts were verified by the staff clinical pathologist.

For determination of anti-*S. neurona* SAG2, 4/3 antibody titers, serum was collected by jugular venipuncture into vacutainer tubes. Blood was allowed to clot at room temperature, the tubes were centrifuged and serum was collected by aspiration and aliquoted. CSF was collected from the LS site following aseptic preparation of the skin. Horses were sedated using xylazine (0.5 mg/kg IV) (AnaSed; Cronus Inc, East Brunswick, NJ) and restrained in a stocks for the procedure. The skin was blocked using 5 mL 2% lidocaine (Lidocaine hydrochloride; VetOne, Boise ID), and a stab incision using a #10 blade made over the LS junction. An 8-inch 18-gauge spinal needle (Mila International, Florence KY) was advanced into the LS space and up to 8 mL CSF collected by gentle aspiration, using the 3-syringe technique. The horses were then euthanized by IV overdose of barbiturate solution (Beuthanasia-D; Schering-Plough, Kenilworth NJ). CSF was then collected immediately from the AO space using a 3.5-inch, 18-gauge spinal needle (Mila International, Florence KY). Two horses (University-owned teaching horses) were not euthanized after the LS tap, and CSF was collected from the spinal cistern using the standing C_1-2_ approach.^3^ Results from standing C_1-2_ collections and AO site collections were analyzed as a group and are referred to as “cranial.” Up to 8 mL of CSF was collected and aliquoted.

Immediately after collection, serum and CSF samples (fresh, chilled) were submitted to a reference laboratory (Equine Diagnostics Solutions, Lexington KY) for determination of anti-*S. neurona* SAG2, 4/3 antibody titers. All samples from an individual horse were run on the same day and using the same ELISA plate. In addition, serum and CSF albumin concentrations were determined using standard methods. The *S. neurona* titer ratio was determined and *S. neurona* AI was calculated from each collection site using standard formulas.^19^ The analyst was blinded to the sample collection site. In addition, a routine CSF analysis was performed on each sample by the Clinical Pathology Laboratory of the Oklahoma State University.

## Abbreviations

AI: antibody index
AO: atlantooccipital
CSF: cerebrospinal fluid
EDTA: ethylene diamine tetraacidic acid
EPM: equine protozoal myeloencephalitis
IQR: interquartile range
LS: lumbosacral
SAG: surface antigen
