# Supplementary material for: Influence of collection site on cerebrospinal fluid test results in horses with equine protozoal myeloencephalitis
Source: J Vet Intern Med. 2026 Jul 8;40(4):aalag135. doi: 10.1093/jvimsj/aalag135 (PMC13345368; doi:10.1093/jvimsj/aalag135)
Supplement: Supplementary_material_aalag135 [file supplementary_material_aalag135.zip › Supplemental Table 2 final v3.docx]

**Supplemental Table 2.** Cell counts for 7 horses with a clinical diagnosis of Equine Protozoal Myeloencephalitis

| **Horse** | **RBC Count Cranial (cells/μl)** | **RBC Count**  **LS**  **(cells/μl)** | **TNCC**  **Cranial**  **(cells/μl)** | **TNCC**  **LS**  **(cells/μl)** | **Lymphocytes**  **Cranial**  **(%)** | **Lymphocytes**  **LS**  **(%)** |
| --- | --- | --- | --- | --- | --- | --- |
| **1** | 0 | 3 | 3 | 2 | 100 | 100 |
| **2** | 0 | 0 | 3 | 3 | 100 | 100 |
| **3** | 0 | 1 | 3 | 3 | 100 | 100 |
| **4** | 0 | 27,770 | 0 | 4,000 | 100 | 27 |
| **5** | 0 | - | 1 | - | 100 | - |
| **6** | 470 | 0 | 6 | 11 | 75 | 85 |
| **7** | 4,708 | 9,333 | 6 | 13 | 29 | 40 |

* - TNCC refers to Total Nucleated Cell Count
