# Supplementary material for: Influence of collection site on cerebrospinal fluid test results in horses with equine protozoal myeloencephalitis
Source: J Vet Intern Med. 2026 Jul 8;40(4):aalag135. doi: 10.1093/jvimsj/aalag135 (PMC13345368; doi:10.1093/jvimsj/aalag135)
Supplement: Supplementary_material_aalag135 [file supplementary_material_aalag135.zip › Supplemental_Table_3_ready to publish.docx]

**Supplemental Table 3.** Diagnostic test results for each horse with Equine Protozoal Myeloencephalitis

| **HORSE** | **Serum Titer*** | **Serum Albumin** | **Cranial CSF Albumin** | **Lumbosacral CSF Albumin** | **Cranial snSAG2 4/3 titer** | **Lumbosacral snSAG2 4/3 titer** | **Cranial titer ratio** | **Lumbosacral titer ratio** | **Cranial AI**** | **Lumbosacral AI**** |
| --- | --- | --- | --- | --- | --- | --- | --- | --- | --- | --- |
|  |  | mg/dl | mg/dl | mg/dl |  |  |  |  |  |  |
| **1** | 500 | 3273 | 48.7 | 78.8 | 5 | 10 | 100 | 50 | 0.67 | 0.83 |
| **2** | 4000 | 3594 | 15.8 | 32.6 | 80 | 160 | 50 | 25 | 4.55 | 4.4 |
| **3** | 2000 | 3434 | 17.4 | 21.6 | 40 | 40 | 50 | 50 | 3.9 | 3.1 |
| **4** | 4000 | 4348 | 44.5 | 137.5 | 40 | 160 | 100 | 25 | 0.98 | 1.3 |
| **5** | 4000 | 5691 | 46.5 | 54.8 | 640 | 1280 | 6.25 | 3.13 | 19.5 | 33.3 |
| **6** | 1000 | 3094 | 32.8 | 36.6 | 160 | 640 | 6.25 | 1.56 | 15.1 | 54.2 |
| **7** | 4000 | 2803 | 47.6 | 84.6 | 640 | 1280 | 6.25 | 3.12 | 9.4 | 10.6 |

* - reciprocal titers reported

** - antibody index

^ - “Cranial” refers to CSF from the atlantooccipital or C1-C2 site
