# Supplementary material for: Influence of collection site on cerebrospinal fluid test results in horses with equine protozoal myeloencephalitis
Source: J Vet Intern Med. 2026 Jul 8;40(4):aalag135. doi: 10.1093/jvimsj/aalag135 (PMC13345368; doi:10.1093/jvimsj/aalag135)
Supplement: Supplementary_material_aalag135 [file supplementary_material_aalag135.zip › Supplemental Table 1 final v3.docx]

**Supplemental Table 1.** Clinical and pathological findings for 7 horses with a clinical diagnosis of Equine Protozoal Myeloencephalitis

| **Horse** | **Clinical Findings** | **Pathologic Findings** |
| --- | --- | --- |
| **1** | Ataxia bilateral hind limbs  Grade 1/5 RH; 2/5 LH  No muscle atrophy | No CNS lesions observed  No organism detected |
|  |  |  |
| **2** | Ataxia  Grade 2/5 LH | No post-mortem. Treated and responded as expected, returned to normal over 4-6 weeks |
|  |  |  |
| **3** | Ataxia bilateral front limbs  Grade 2/5 | No CNS lesions observed  No organism detected |
|  |  |  |
| **4** | Ataxia bilateral hind limbs.  Grade 2/5 RH; 3/5 LH  Severe hindlimb weakness; “Crab walking” left side  Mild left gluteal atrophy, decreased placing L front | Spinal cord (C2, C4), mild focal perivascular lymphocytosis  Mild focal lymphocytosis C4 dura  No organisms detected  Pituitary Adenoma |
|  |  |  |
| **5** | Ataxia bilateral hindlimbs  Grade 3/5 | Lymphohistiocytic perivasculitis and meningoencephalitis, multifocal (cerebrum, cerebellum, brainstem, spinal cord C1, L1 and T2)  T16, L1 axonal degeneration  Eosinophilic perivasculitis L1  No organisms observed |
|  |  |  |
| **6** | Ataxia bilateral hind limbs  Grade 2/5  Masseter atrophy left  Absent slap test left side | Lymphohistiocytic perivasculitis, multifocal (brainstem, spinal cord C2 and C5)  No organism detected |
|  |  |  |
| **7** | Ataxia bilateral hind limbs. Grade 2/5 LH; 3/5 RH. Severe weakness RH; crab-walking right side | No post-mortem. Treated and responded as expected, returned to normal over 4-6 weeks |

LH – Left Hind; RH – Right Hind ; CNS – central nervous system
